# Advances in epidemiological status and pathogenesis of dental fluorosis

**DOI:** 10.3389/fcell.2023.1168215

**Published:** 2023-05-05

**Authors:** Kaiqiang Zhang, Zhenfu Lu, Xiaoying Guo

**Affiliations:** ^1^ Department of Preventive Dentistry, School of Stomatology, China Medical University, Shenyang, Liaoning, China; ^2^ Department of Environmental Health, School of Public Health, China Medical University, Shenyang, Liaoning, China

**Keywords:** dental fluorosis, pathogenesis, epidemiology, signal pathway, molecular mechanism

## Abstract

Fluoride is commonly consider as a “double-edged sword” because low consumption of fluoride can effectively prevent dental caries, but excessive consumption of fluoride can cause fluorosis. Dental fluorosis (DF) is a characteristic feature of fluorosis in the oral cavity that is manifested as tooth color changes and evident enamel defect. Presently, the pathogenesis of DF remains unclear. Herein, we have summarized the research progress in the pathogenesis and mechanism of DF in the past 5 years.

## 1 Introduction

Fluoride is ubiquitous and is one of essential trace elements that are required by humans. It has a strong affinity for hard tissue and can easily be deposited on the teeth and bones. Long-term exposure to a high concentration of fluoride before the age of 7 year can cause fluorosis, which is mainly manifested as dental fluorosis (DF) in the oral cavity. The characteristic manifestation of DF includes tooth color changes and enamel defects; however, its pathogenesis remains unclear. DF is found worldwide, and there are significant regional differences in the prevalence rate owing to the differences in interfering factors. Through the development of modern stomatology, fluoride is widely used for preventing dental caries. However, DF caused by excessive fluoride intake has gained increasing public attention at the same time. Previous studies have shown that the symptoms of DF are influenced by a variety of factors, including the amount of fluoride intake, age, living environment, and genetics of patients (Abanto Alvarez et al., 2009). During enamel maturation, excessive fluoride accumulation can induce oxidative stress and lead to damage caused by ROS. To mitigate this damage, autophagy is activated, initiating an adaptive defense response, and inducing ameloblast apoptosis by activating the caspase pathway (Suzuki et al., 2015). In addition, fluoride can cause upregulation of Ca^2+^ in ameloblast, which triggers an endoplasmic reticulum stress response and induces apoptosis through the GRP78-CHOP-Bcl2 pathway (Sharma et al., 2008). Herein, we have summarized the research progress in the pathogenesis and mechanism of DF in the past 5 years.

## 2 Epidemiological status of DF

Fluoride can prevent dental caries; however, excessive fluoride can cause DF. Fluorosis is also known as endemic fluorosis (EF), and it is mainly caused by consuming items that are ubiquitous in the natural environment, such as drinking water, brick tea, or coal with a high fluoride content. Another type of fluorosis is caused by excessive use of fluoride toothpaste, fluoride-containing beverages, and iatrogenic fluorosis, and its epidemiological characteristics are not clearly defined. For consistency, we have referred to it as other types of fluorosis (OTF). The outcomes of DF are also completely different due to the different causes of the disease. OTF rarely causes DF with Thylstrup—Fejerskov index > 2, with minor enamel defects and tooth color change, and the effect on the appearance of teeth is generally accepted by most patients (Doumit and Doughan, 2018; Saldarriaga et al., 2021c; Restrepo et al., 2022). However, EF can cause severe DF with distinct enamel defects, which has a considerable impact on the quality of life of patients; therefore, active prevention and treatment are encouraged (Mohd Nor et al., 2021; Rani et al., 2022).

The prevalence of DF varies greatly owing to different interfering factors. From the point of view of diet, excessive fluoride in drinking water is considered as the main reason for the high prevalence of DF (Kumar et al., 2018). The widespread use of foods, such as fluorinated beverages and fluorinated salt, is further increasing the incidence of DF (Aguilar-Diaz et al., 2017), which has become one of the major reasons for the increase in DF cases. In the group of children before the age of 7, calcium and vitamin intake can affect the incidence of DF. A survey on children with fluorosis living in areas with high fluoride content showed that the dietary calcium intake of the children was negatively associated with the incidence of DF. When the calcium intake is high (>520 mg/day), the symptoms of fluorosis were effectively prevented or alleviated (Tefera et al., 2022). Another report suggested that the intake of α-carotene, β-carotene, lutein, lycopene, and carotenoids is also negatively associated with DF (Li et al., 2022b); therefore, their consumption can alleviate DF symptoms.

From a socio-economic point of view, social class is a major reason for the difference in morbidity. Morbidity is often distinct in high fluoride areas. Financially well-off people can purchase drinking water with normal fluoride contents or install water purification systems, whereas financially challenged people might only be able to afford natural water with high fluoride contents (Patel et al., 2017). There is also a difference in the incidence of DF between rural and urban areas in China owing to the difference in the various ways of obtaining water, which is related to the construction and popularization of infrastructure including tap water networks (Zhou et al., 2018). Infrastructure construction is closely related to local economic level.

The effect of gender on the incidence of DF has not been investigated yet. An epidemiological survey of 929 adolescents in Northern Colombia revealed that the prevalence of DF did not differ significantly between the two genders and that it had no significant effect on the incidence (Saldarriaga et al., 2021b), which is consistent with the results of another survey of 1,044 adolescents in India (Verma et al., 2017). However, notably, economically underdeveloped areas and the unequal status of different genders can cause differences in the diet and living habits of people, which may contribute to the differences in its prevalence.

In terms of the living environment, there is an increase in the prevalence of people who is in the plateau. These people have the habit of drinking brick tea and using coal or wood containing fluoride to make food, which leads to the spread of fluoride from the fuel into the air and its absorption by the human respiratory tract (Saldarriaga et al., 2021a). In areas with high fluoride contents in rock or soil, fluoride leaching can occur, resulting in groundwater pollution. However, owing to the different natural solubility of water, temperature, mineral interference, and soil acidity of different regions, the absorption of fluoride by the human body is also different (Xu et al., 2017).

From a genetic point of view, studies suggest that genetic factors are one of the reasons for differences in the susceptibility to fluoride in the same exposure environment. A study of 676 adolescents showed that the content of mitochondrial DNA (mtDNA) in patients with DF was significantly lower than that in patients without DF, and the negative correlation between mtDNA levels and DF in boys was higher than that in girls (Zhou et al., 2019). Furthermore, a study on the polymorphism of the distal-less homeobox-1 (DLX1), distal-less homeobox-2 (DLX2), matrix metallopeptidase-13, tissue inhibitor of metalloproteinase-1, and tissue inhibitor of metalloproteinase-2 genes found that DF is more common in people of the African descent than among Caucasians and that the polymorphisms of TIMP1, DLX1, and DLX2 genes may be associated to their phenotypes (Kuchler et al., 2017). Charone et al., 2019 reported that polymorphisms in Ameloblastin (Ambn), Collagen Type XIV Alpha 1 Chain, and Matrix Metallopeptidase 20 (MMP-20) were related to susceptibility to DF through genotyping of 17 genetic polymorphisms such as Amelogenin X-Linked (Amelx) and Ambn. In addition to the abovementioned results, the genetic polymorphisms of ESR1 (Dalledone et al., 2019), Superoxide Dismutase 2 (Du et al., 2022), Collagen Type I Alpha 2 Chain (Jarquin-Yneza et al., 2018), AMBN, Tuftelin Interacting Protein 11, and Tuftelin 1 (Kuchler et al., 2018) can lead to differences in the incidence of DF.

## 3 Research progress in the pathogenesis of DF

Previous studies on the pathogenesis of DF mainly suggested that high fluoride affects the secretion of the enamel matrix proteins, such as AMELX, AMBN, and ENAM, and the activity of matrix metalloproteinases, such as KLK-4 and MMP20 (Houari et al., 2019). This results in the abnormal development of enamel, which involves physiological processes such as endoplasmic reticulum stress, oxidative stress, mitochondrial damage, DNA damage, and apoptosis. Recently, phosphoinositide-3-kinase (PI3K)/AKT signal pathway, autophagy pathway, Ca^2+^ and mitochondrial homeostasis, extracellular signal-regulated kinase (ERK) pathway, and the regulation of microRNA have gradually become the focus of research to investigate the pathogenesis of DF (Zhao et al., 2016; Deng et al., 2020; Zhao et al., 2022).

### 3.1 Role of the PI3K/AKT pathway in DF development

p53, an important effector molecule of the PI3K/AKT pathway, interacts with AKT via the murine double minute 2 (MDM2) pathway, responds to various genotoxic damage and cellular stress, and affects many important cellular processes, such as proliferation, DNA repair, apoptosis, autophagy, metabolism, and cell migration. Studies (Deng et al., 2020) showed that CREB-binding protein (CBP)/p300, acetyltransferase p300/CBP-associated factor, and Tip60, as upstream signal molecular pathways, promoted fluoride-mediated p53 acetylation and increases p53 acetylation in mouse ameloblasts (LS8), cell growth inhibition, apoptosis, and mitochondrial damage. After inhibiting p53 acetylation, cell growth inhibition was reversed and the levels of cleaved caspase-3, the DNA damage marker *γ* H2A histone family member X, and the mRNA ratio of B-cell leukemia 2 (Bcl-2)-associated X protein/Bcl-2 decreased significantly. Suzuki et al. (2018) also showed that excessive fluoride mediated p53 deacetylation by activating the sirtuin 1 pathway, mitigated cell growth inhibition, mitochondrial damage, DNA damage and apoptosis. Fujiwara et al. (2021) found that a high dose of fluoride could activate the p53-p21 pathway and curcumin inhibited fluoride-mediated cleaved caspase-3 by phosphorylating Akt at Thr308, thus reducing cell damage. Akt, the core molecule of the PI3K/AKT pathway, can effectively decrease fluoride-induced apoptosis and inhibit DNA damage in ameloblasts. Other studies have shown that MDM2-mediated p21 proteasome degradation and the subsequent decrease in p21 phosphorylation are involved in fluoride-induced apoptosis (Deng et al., 2019), suggesting that inhibiting p21 degradation may be a potential therapeutic target to reduce fluoride toxicity. Moreover, AKT can regulate cell-cycle- and bone-differentiation-related factors via the downstream forkhead box protein O (FOXO) pathway (Li et al., 2018). Studies have shown that fluoride significantly reduced proliferating cell nuclear antigen, runt-related transcription factor 2 (RUNX2), and matrix metalloproteinase 9 levels in ameloblasts. The levels of these proteins are closely related to enamel mineralization. The FOXO1 pathway decreased the levels of Runx2 mRNA and protein and KLK-4 in ameloblasts after fluoride intervention, which further affected enamel mineralization (Li et al., 2022a). Runx2 can also regulate Dlx2 via the miR-185-5p pathway to inhibit ameloblast differentiation and affect Amelx and Enam expression (Chang et al., 2017). To summarize, the PI3K/AKT pathway with AKT as the core molecule plays a critical regulatory role in DF development.

### 3.2 Role of autophagy in DF development

Zhao et al. (2022) established an *in vitro* model using LS8 cell line and induced autophagy. They found that NaF induced autophagy in LS8 and autophagy-related 5 and autophagy-related 7 were important molecules involved in NaF-induced autophagy. Blocking NaF-induced autophagy could restore the ameloblast activities, and NaF-induced apoptosis could be reversed by inhibiting early autophagy. Yao et al. showed considerable changes in microtubule-associated protein light chain (LC) 3, LC3II, and sequestosome 1 (p62) expression in NaF-treated ameloblasts (Yao et al., 2023). Moreover, the significantly improved growth of LS8 cells treated with NaF and rapamycin, an autophagy inhibitor, and the significantly decreased expression of p62 suggested that the fluoride-induced impairment of autophagosome degradation might damage ameloblasts. Rapamycin may alleviate this damage by reducing p62 expression.

### 3.3 Role of Ca^2+^ homeostasis in DF development

Ca^2+^ plays a vital role in maintaining homeostasis and signal transmission. Recent studies have shown that it can affect the function of ameloblasts in the following ways: 1) Fluoride mediates the release of Ca^2+^ from the endoplasmic reticulum of ameloblasts via inositol 1,4,5-trisphosphate receptors (Aulestia et al., 2020). Studies have shown that in a high-fluoride environment, compared with the control group, Ca^2+^ can increase the activity of KLK-4, improve the inhibitory effect of fluoride on ameloblast growth, and reduce apoptosis, which is related to the activation of the protein kinase R-like endoplasmic reticulum kinase–eukaryotic initiation factor 2 α-activating transcription factor 4–C/EBP homologous protein pathway (Liu et al., 2021). 2) The PI3K/AKT pathway is involved in the intracellular calcium signal pathway of ameloblasts. With an increase in calcium concentration, the levels of KLK-4 and amelotin in ameloblasts increased significantly, whereas the levels of PI3K, AKT, p-AKT, and FOXO3 decreased significantly after calcium treatment (Gao et al., 2020). 3) Fluoride also affects mitochondria, one of the important calcium stores in ameloblasts. After fluoride treatment, the calcium-uptake ability of mitochondria, membrane potential, and the ATP turnover rate of ameloblasts decreased, and morphological changes in mitochondria were observed under a transmission electron microscope (Aulestia et al., 2020), which suggested that fluoride affected ameloblast homeostasis by affecting mitochondrial functions. 4) Stromal interaction molecule (STIM) mutation affects the Ca^2+^ release channel (calcium release-activated channel) and storage channel [Store-operated calcium entry ([SOCE)]. Miriam Eckstein (Eckstein et al., 2017), using animal models, showed that Stim1/2K14^cre^ mice exhibited insufficient enamel mineralization, decreased Ca^2+^ content, mitochondrion mislocalization, increased reactive oxygen species levels, decreased mitochondrial functions, and abnormal mitochondrial morphology, suggesting that SOCE loss in ameloblasts affected cell functions and enamel mineralization. Another study showed that in STIM1-and STIM1/2-conditioned gene-knockout mouse incisors, the alternating frequency cycles of mature ameloblasts at smooth and wrinkled ends were abnormal, indicating that SOCE was essential for normal enamel mineralization, and STIM1 played a key role in enamel maturation (Furukawa et al., 2017).

### 3.4 The role of ERKs in DF development

The triple kinase cascade of the mitogen-activated protein kinase signal transduction pathway is involved in many cellular processes, from extracellular stimulation to the corresponding biological effects on cells (Nakamura et al., 2021). Enamel formation is highly dependent on kinases, and MAPK phosphatase 1 (MKP-1) plays a key role in regulating ERK-associated kinases. Fluoride exposure inhibited p-MEK and p-ERK1/2, followed by an increase in MKP-1 levels in a dose-dependent manner. Further, studies showed that MKP-1, a negative regulator of fluoride-induced p-ERK1/2 signal transduction, downregulated CREB, c-myc, and Elk-1 at high doses of fluoride (Zhao et al., 2021). MKP-1 can bind and dephosphorylate ERK and downstream p38 and c-Jun N-terminal kinases (JNK). JNK levels in fluoride-induced ameloblasts are closely associated with apoptosis (Papa et al., 2019), thus, changing the pathway also plays an important role in enamel formation.

### 3.5 Research progress of microRNAs (miRNAs) in DF development

miRNAs can play a regulatory role by destroying and inhibiting the stability and translation of target mRNAs; thus, they have potential clinical applications. Luo et al. (2022) found that the expression of miR-296-5p in LS8 cells treated with excessive fluoride decreased, which further enhanced autophagy and participated in autophagy regulation by activating the AMP-activated protein kinase/Unc-51-like autophagy activating kinase 1 pathway. MiR-185-5p participates in the regulation of ameloblast development and differentiation via the Dlx2 pathway, and miR-31 regulates the proliferation of dental epithelial cells by targeting Satb2 (Chang et al., 2017; Tian et al., 2020). A study on the role of miRNA in regulating circadian rhythm during enamel development showed that 191 known miRNAs showed differential expression (Nirvani et al., 2019). The above studies suggest that the potential roles of miRNAs in ameloblast differentiation and functions need to be thoroughly investigated. miRNA is involved in enamel formation, which consists of aspects including cell development, differentiation, and cycle. However, these topics need to be researched further for better clarification and understanding.

## 4 Outlook and future perspectives

DF is a global problem, and its epidemic area is widely distributed but uneven. Though its cause is clear, the ways of prevention vary because of different interference factors. From the epidemiological trend, it is foreseeable that the number of DF patients caused by substandard drinking water will gradually decrease, but the number of DF patients caused by iatrogenic or excessive intake of fluoride to prevent dental caries will gradually increase. The characteristics of enamel development indicate that DF cannot heal itself once it is formed. Therefore, during the critical period of enamel formation in children before 7 years old, fluoride intake should be strictly controlled. Fluoride intake may damage hard tissues and affect the intelligence of children (Yu et al., 2018) and exerts potential neurotoxic and reproductive toxic effects (Adedara et al., 2017; Sm and Mahaboob Basha, 2017), suggesting that fluoride may affect the differentiation and development of other organs in addition to hard tissues. From the perspective of cell biology, enamel development is generally divided into four stages, and ameloblasts play different roles in each stage (Sarkar et al., 2014); hence, simulating the whole process of enamel development in a single traditional cell culture model is challenging. Current studies have great limitations, and three-dimensional cell culture technology will provide new ideas for future research. With a gradual increase in research, DF pathogenesis is becoming clearer. However, studies on the role of miRNAs, long non-coding RNAs, circular RNAs, and exosomes in DF are unavailable, which need further exploration. Mitochondria are an important intracellular calcium pool playing a role in maintaining intracellular calcium homeostasis. Moreover, mitochondrial DNA and energy supply mechanisms are an important research direction. With increasing research, more ways to prevent and treat DF and reduce the harm caused by it to the population will be available.
